# The Diagnostic Performance of an Extended Ultrasound Protocol in Patients With Clinically Suspected Giant Cell Arteritis

**DOI:** 10.3389/fmed.2021.807996

**Published:** 2022-01-18

**Authors:** Johan Skoog, Christina Svensson, Per Eriksson, Christopher Sjöwall, Helene Zachrisson

**Affiliations:** ^1^Department of Clinical Physiology and Health, Medicine and Caring Sciences, Linköping University, Linköping, Sweden; ^2^Division of Inflammation and Infection/Rheumatology, Department of Biomedical and Clinical Sciences, Linköping University, Linköping, Sweden

**Keywords:** giant cell (temporal) arteritis, color duplex ultrasound, large vessel vasculitis, diagnostic imaging, IMT (intimal medial thickness)

## Abstract

**Objective:**

To evaluate the diagnostic performance of an extended ultrasound protocol in patients referred under the suspicion of giant cell arteritis (GCA).

**Methods:**

Consecutive patients with suspected GCA were examined with an extended color duplex ultrasound (CDU) protocol during a period of 2 years. The extended CDU protocol included temporal, axillary, subclavian, brachiocephalic, and carotid arteries. The reference was clinically diagnosed GCA, confirmed after ≥6-month follow-up. Hypo- or medium-echogenic, circumferential, homogenous wall thickening, and/or a positive compression sign in temporal arteries, were regarded as typical signs of arteritis.

**Results:**

Of the eligible 201 patients, 83 (41%) received a clinical GCA diagnosis at follow-up ≥6 months post CDU examination. Among these cases, 48 (58%) demonstrated inflammation solely in temporal arteries, 8 (10%) showed abnormalities restricted to extra-cranial vessels, and 23 (28%) patients displayed inflammatory changes in both temporal and extra-cranial arteries. Color duplex ultrasound of temporal arteries yielded a diagnostic sensitivity and specificity [95% confidence intervals (CI)] of 86% (76–92%) and 99% (95–99%), respectively. By adding axillary artery examination, the sensitivity increased to 92% (83–97%) while the specificity remained unchanged. Further, inclusion of subclavian artery marginally increased the sensitivity by 1%. Finally, by also including brachiocephalic and common carotid arteries resulted in a sensitivity of 95% (88–99%) and a specificity of 98% (94–99%).

**Conclusions:**

Color duplex ultrasound examination demonstrated a high accuracy in diagnosing patients both with cranial and extra-cranial GCA. Further examination of brachiocephalic and common carotid arteries can increase the sensitivity without affecting the specificity when temporal and axillary findings are indecisive. Finally, the extended CDU protocol allows measurement of the general burden of inflammation, which could be relevant for future monitoring purposes.

## Introduction

Giant cell arteritis (GCA) is a systemic inflammatory disease targeting mainly large-sized arteries (1). The classical form of GCA is characterized by headache, scalp tenderness, jaw claudication, and visual loss which all manifests cranial symptoms of GCA (2). However, it is not uncommon that patients with GCA demonstrates extra-cranial vessel inflammation accompanied by diffuse inflammatory symptoms in the absence of headache, visual symptoms, or jaw claudication. Vascular imaging has shown involvement of the aorta and its major branches in up to 83% of such cases (3). Rapid diagnosis and treatment are important to reduce the risk of complications (4, 5). Temporal artery biopsy (TAB) has been considered the gold standard test for the diagnosis of GCA but newer recommendations support that color duplex ultrasound (CDU), if available, should serve as the first diagnostic tool in patients presenting with predominantly cranial GCA (6, 7). EULAR recommends ultrasound of temporal and axillary arteries as the primary imaging test, as examination of axillary arteries may be helpful in patients with suspected GCA who display negative or indecisive temporal artery ultrasound (6). However, it has been suggested that inclusion of axillary arteries only slightly increase the diagnostic sensitivity (8), whereas this examination may fail to identify large-vessel GCA. It has therefore been proposed that additional examination of the subclavian artery may facilitate the diagnosis (9). Nevertheless, the diagnostic benefit of evaluating different vascular beds with CDU in addition to cranial arteries remains largely unknown (10). We have previously developed an extended ultrasound protocol for detection of vessel wall inflammation (11). The aim of this study was to evaluate the diagnostic performance of an extended CDU examination, which in addition to temporal and axillary arteries, also includes subclavian, brachiocephalic and carotid arteries. This was performed as the first-line investigation in patients with suspected GCA as compared to current recommendations of temporal and axillar ultrasound alone. It was hypothesized that our extended ultrasound protocol would increase the diagnostic sensitivity for GCA without substantially affecting the specificity.

## Materials and Methods

### Study Population

This retrospective study comprised all patients examined with CDU at the Department of Clinical Physiology, Linköping, Sweden, between January 2018 and December 2019 due to clinically suspected GCA. Practically all patients examined with CDU presented with elevated levels of C-reactive protein (CRP) combined with unspecific inflammatory symptoms where arteritis not could be excluded. Some patients had fever, weight loss, morning stiffness, and tiredness, whereas others presented symptoms more specific for cranial arteritis, such as headache, jaw claudication, amaurosis fugax, or proximal extremity pain suggestive of polymyalgia rheumatica (PMR) (Table 1).

**Table 1:** Patients' characteristics and comparison between patients with and without giant cell arteritis (GCA).

**Patients' characteristics**	**Patients with GCA (*n* = 83)**	**Patients without GCA (*n* = 118)**	***p*-Values**
Age, median (range) years	76 (51–94)	71 (37–98)	0.0001
Female, *n* (%)	60 (72)	78 (66)	0.44
Smoking, *n* (%)	10 (12)	4 (3)	0.024
Clinical characteristics, *n* (%)			
New headache	60 (72)	69 (58)	0.052
Jaw claudication	29 (35)	15 (13)	0.0002
Reduced or lost vision	16 (19)	11 (9)	0.058
Double vision	5 (6)	1 (1)	0.084
Temporal artery abnormalities^a^	46 (58)	29 (25)	<0.0001
Myalgia, upper extremity	27 (33)	45 (38)	0.46
Myalgia, lower extremity	15 (18)	41 (35)	0.011
Joint pain	18 (22)	36 (31)	0.20
Joint swelling	4 (5)	19 (16)	0.014
Morning stiffness	13 (16)	35 (30)	0.029
Fatigue	77 (93)	96 (81)	0.023
Loss of appetite	22 (27)	24 (20)	0.31
Weight loss >2 kg	17 (21)	17 (14)	0.34
Temp >38.5°C	5 (6)	15 (13)	0.15
Laboratory findings			
ESR, mm/h, median (range)	75 (6–119)	64 (1–115)	0.024
CRP, mg/L, median (range)	53 (5–338)	35 (2–335)	0.004
Comorbidity, *n* (%)			
Hypertension	48 (58)	69 (59)	1.0
Diabetes mellitus	11 (13)	21 (18)	0.44
Dyslipidemia	27 (33)	37 (31)	0.88
Myocardial infarction	5 (6)	12 (10)	0.44
Cerebrovascular disease	6 (7)	18 (15)	0.12
Peripheral artery disease	2 (2)	3 (3)	1.0
Polymyalgia rheumatica	12 (15)	26 (22)	0.20

*Erythrocyte sedimentation rate (ESR) and C-reactive protein (CRP) were measured before initiation of high-dose glucocorticoid treatment.*

a *Tenderness, pain, swelling, or decreased pulsations*.

The diagnosis of GCA was based on a model proposed by Czihal et al. using both CDU and clinical parameters (12). Patients were classified as GCA if the 1990 American College of Rheumatology (ACR) criteria were satisfied (13), and/or if patients had the typical ultrasound picture of arteritis characterized by hypo- or medium echogenic, homogenous, circumferential wall thickening combined with increased levels of CRP and/or erythrocyte sedimentation rate (ESR), and good clinical response to corticosteroids.

At least 6 months after the CDU examination, two experienced rheumatologists (PE and CSj), not responsible for clinical care of the patients, reevaluated all data, judged the final clinical diagnosis of arteritis, and excluded other diagnoses explaining inflammatory disease. In order to achieve this, data were collected from a digital medical record system including all parts of the health care system from the catchment area. The use of a 6-month clinical follow-up as the reference is commonplace for studies of GCA (10, 14).

Patients with (1) GCA diagnosis prior to the CDU examination; (2) follow-up CDU; (3) deceased or migrated within 6 months after the CDU; (4) high doses steroids >2 month preceding CDU, were excluded.

### CDU Assessment

**Figure 1:**
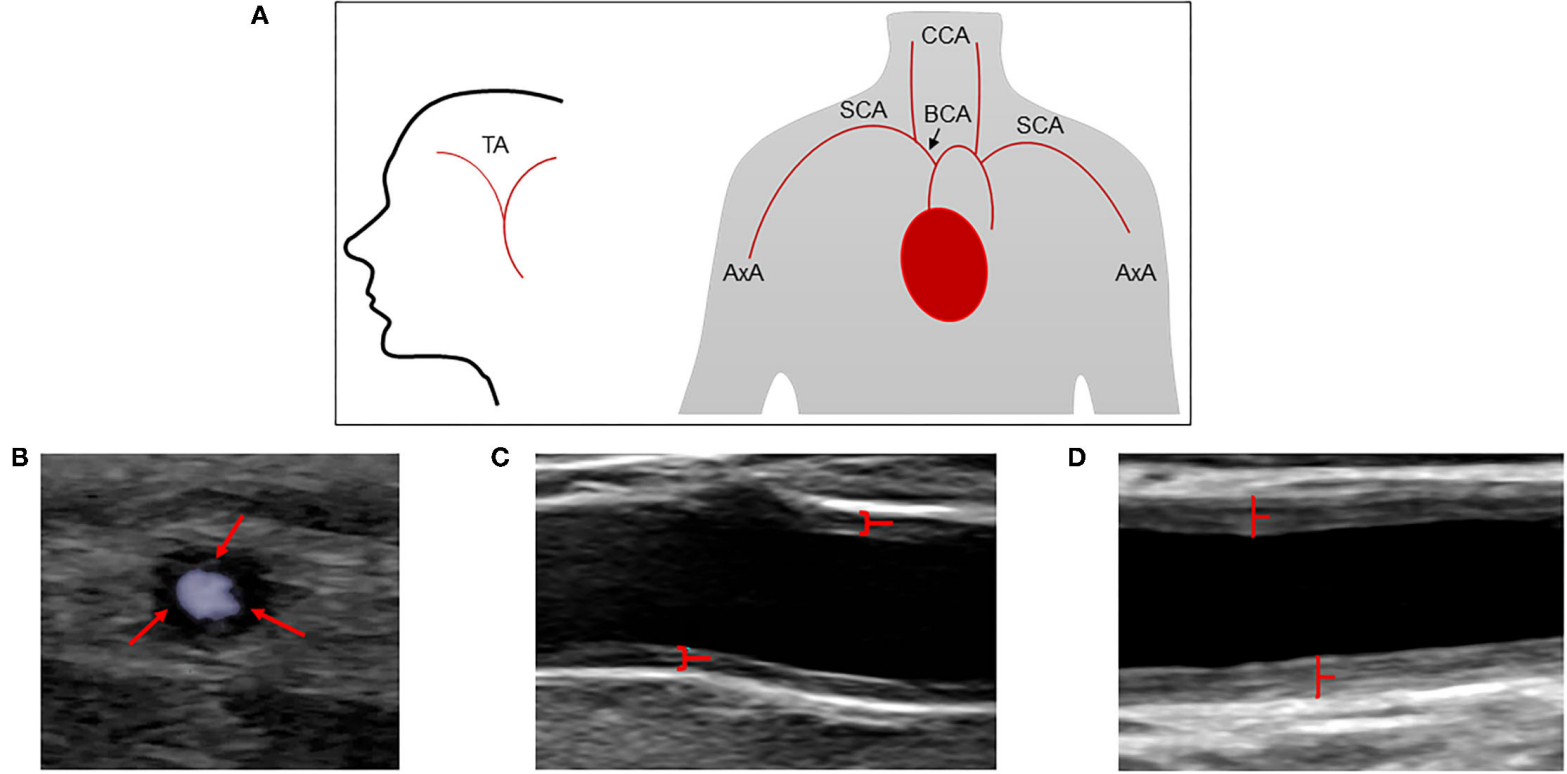
Extended CDU protocol and assessment of cranial and extra cranial vessels by CDU. **(A)** Schematic illustration of the extended CDU protocol. **(B)** Increased circumferential low echogenicity vessel wall thickness in the common temporal artery, often described as halo sign (red arrows). **(C)** Increased circumferential vessel wall thickness (medium echogenicity) in near and far wall of the axillary artery (red marked). **(D)** Marked increased circumferential vessel wall thickness (medium echogenicity) in near and far wall of the common carotid artery (red marked). TA, temporal artery; AxA, axillary artery; SCA, subclavian artery; BCA, brachiocephalic artery; CCA, common carotid artery.

A GE Logic E9 and E10 US system (LOGIQ E9 and E10 XDclear 2.0 General Electric Medical Systems US, Wauwatosa, WI, USA) with linear transducer L2-9 MHz and high frequency hockey stick transducer L8-18i were used for ultrasound measurements. The three branches of the temporal artery (common superficial artery, parietal, and frontal branches) were examined, as well as axillary, subclavian, brachiocephalic, common carotid, internal, and external carotid artery. Both sides were investigated, and the CDU protocol as well as measuring principles are shown in Figures 1A–D. The protocol has previously been described in detail (11, 15, 16). Color duplex ultrasound examinations were classified as positive if signs of inflammation were observed in at least one vessel. Hypo or medium echogenic, circumferential, homogenous wall thickening, and/or a positive compression sign of temporal arteries, were regarded as typical signs of arteritis (17–19). The intima-media thickness (IMT) was measured in all investigated vessels, with the exception of temporal arteries. Plaques were defined as focal areas in the vessel wall where IMT showed increase of either 0.5 mm or 50% compared to the IMT in the adjacent wall. Four experienced vascular technologists performed the CDU examinations as part of a standardized routine examination. One vascular technologist and three physicians performed the final interpretation of the CDU examinations. Complicated CDU images were reviewed according to clinical routine.

### TAB

The local pathologists reported TAB positive or negative for GCA as part of standard practice. Temporal artery biopsy was defined positive if the sample showed vasculitis characterized by a high proportion of mononuclear cell infiltration or granulomatous inflammation, typically with multinucleated giant cells (13).

### Statistical Evaluation

Data are presented as numbers and percentages or median with min and max value or the 25^th^ and 75^th^ percentile. Differences between patients with or without GCA were evaluated with the Mann–Whitney *U*-test. Categorical variables were tested with Fisher's exact test. The sensitivity and specificity (95% CI) of CDU were calculated using the clinical GCA diagnosis after 6 months as reference. Further, positive and negative prediction values as well as accuracy were calculated for the extended CDU protocol. Statistical analyses were carried out using SPSS 27.0 for windows (IBM, Armonk, NY, USA). *p*-Values < 0.05 were considered significant.

### Ethical Considerations

The study was performed according to the declaration of Helsinki, and the study protocol was approved by the Regional Ethical Board in Linköping (ref. 2013/33-31).

## Results

### Outcomes

**Figure 2:**
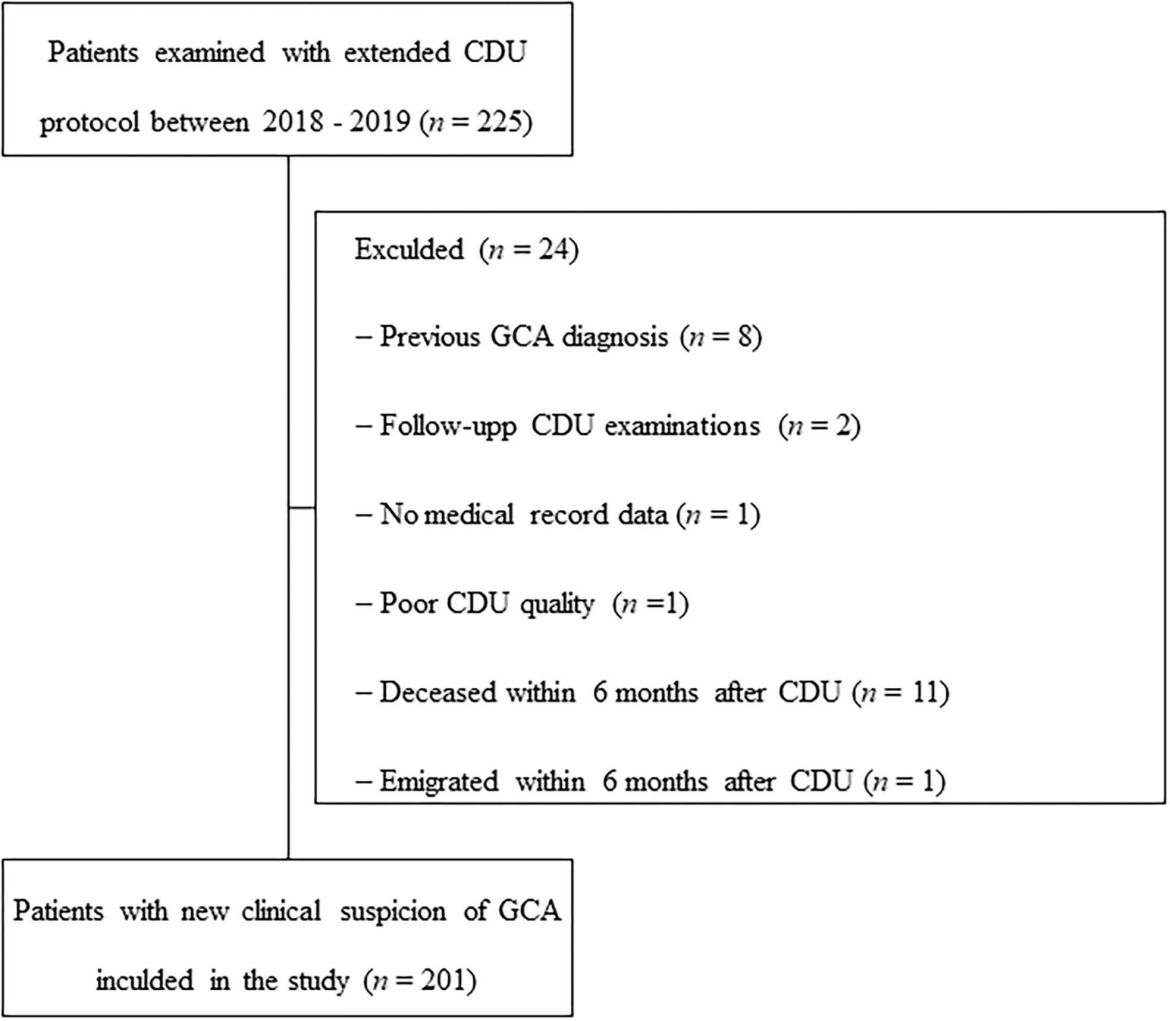
Flowchart of patient inclusion.

In total, 225 cases were examined with an extended ultrasound protocol during 2018–2019, and 201 of these subjects were included in the study (Figure 2). Of the included 201 patients with suspected GCA, the median age (range) was 73 years (37–98) and 138 (69%) were females. An evaluation after ≥6 months yielded a clinical diagnosis of GCA in 83 (41%) patients. Sixty-one of the 83 patients diagnosed with GCA fulfilled the ACR criteria (73%). Diagnoses of the non-GCA group are shown Supplementary Table 1. Baseline characteristics of those with and without clinical diagnosis of GCA are detailed in Table 1. Patients with GCA were more likely to have jaw claudication, temporal artery abnormalities, symptoms of fatigue, and higher levels of CRP and ESR compared to the non-GCA group. TAB was performed in 51 cases (25%), in which 27 (53%) were negative and 24 (47%) were positive. Consistent data from both TAB and CDU, not supporting a diagnosis of GCA, were seen in 14 patients, whereas consistent data compatible with a diagnosis of GCA from both modalities were seen in 20 patients. Thus, 13 patients displayed inflammatory changes during CDU examination but normal TAB, and 4 patients vice versa.

### Extended CDU Protocol

**Figure 3:**
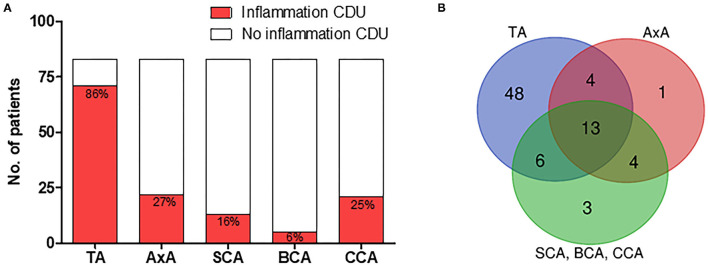
CDU finding in patients with GCA. **(A)** Localization of CDU detected vessel wall inflammation in patients with clinical GCA diagnosis. **(B)** Temporal, extra-cranial, and combined temporal and extra-cranial vessel wall inflammation in patients with clinical GCA diagnosis. TA, temporal artery; AxA, axillary artery; SCA, subclavian artery; BCA, brachiocephalic artery; CCA, common carotid artery.

Figures 3A,B shows the localization of CDU abnormalities in the 83 patients with GCA. The temporal artery was affected in 71 (86%), axillary artery in 22 (27%), subclavian artery in 13 (16%), brachiocephalic artery in 5 (6%), and common carotid artery in 21 (25%) of the patients (Figure 3A). In these patients, 48 (58%), demonstrated signs of vessel wall inflammation only in temporal arteries, 8 (10%) showed abnormalities restricted to extra cranial vessels and 23 (28%) patients displayed inflammatory changes in both temporal and extra cranial arteries (Figure 3B). Of the 118 patients not diagnosed with GCA, two patients displayed abnormalities during CDU examination. The temporal artery was affected in one patient, and the other demonstrated atypical inflammatory signs in common carotid artery and brachiocephalic artery, i.e., an asymmetric, hypo to medium echogenic, and homogenous vessel wall thickening of inflammatory type. The former was diagnosed with headache of unknown origin and the latter with chronic myelomonocytic leukemia.

Patients with GCA displayed a significantly higher IMT in large vessels bilaterally compared to the non-GCA group, with the exception of the brachiocephalic artery (Table 2). Atherosclerotic plaques in neck arteries were common in both groups and only 13 (16%) in the GCA group and 18 (15%) in the non-GCA group showed no plaques. The most frequent localizations of atherosclerotic plaques were in the carotid bifurcation where 41 (49%) of patients with GCA and 50 (42%) of patients with no GCA had plaque formation. No differences in localization or frequency of plaques were seen between the groups (Table 2).

**Table 2:** IMT and atherosclerotic plaque in patients with and without giant cell arteritis (GCA).

**Variable**	**Patients with GCA (*n* = 83)**	**Patients without GCA (*n* = 118)**	***p*-Values**
IMT, right			
AxA, mm median (IQR)	0.70 (0.60–1.00)	0.60 (0.50–0.70)	0.007
SCA, mm median (IQR)	0.70 (0.60–1.00)	0.70 (0.60–0.80)	0.007
BCA, mm median (IQR)	1.00 (1.25–1.45)	1.1 (0.90–1.50)	0.63
CCA, mm median (IQR)	0.80 (0.70–1.00)	0.80 (0.60–0.90)	0.026
IMT, left			
AxA, mm median (IQR)	0.70 (0.60–0.92)	0.60 (0.50–0.70)	0.0001
SCA, mm median (IQR)	0.70 (0.60–1.00)	0.60 (0.50–0.80)	0.003
CCA, mm median (IQR)	0.80 (0.70–1.10)	0.70 (0.60–0.90)	0.003
Atherosclerotic plaque			
No plaque, *n* (%)	13 (16%)	18 (15%)	1.0
AxA, *n* (%)	9 (11%)	7 (6%)	0.29
SCA, *n* (%)	2 (2%)	1 (1%)	0.57
ICA, *n* (%)	2 (2%)	1 (1%)	0.57
ECA, *n* (%)	–	2 (2%)	0.51
Carotid bifurcation, *n* (%)	41 (49%)	50 (42%)	0.39
≥3 vessels, *n* (%)	21 (25%)	36 (31%)	0.43

*IQR, interquartile range; GCA, giant cell arteritis; IMT, intima media thickness; AxA, axillary artery; SCA, subclavian artery; BCA, brachiocephalic artery; CCA, common carotid artery; ICA, internal carotid artery; ECA, external carotid artery*.

### Diagnostic Performance of an Extended CDU Protocol

**Figure 4:**
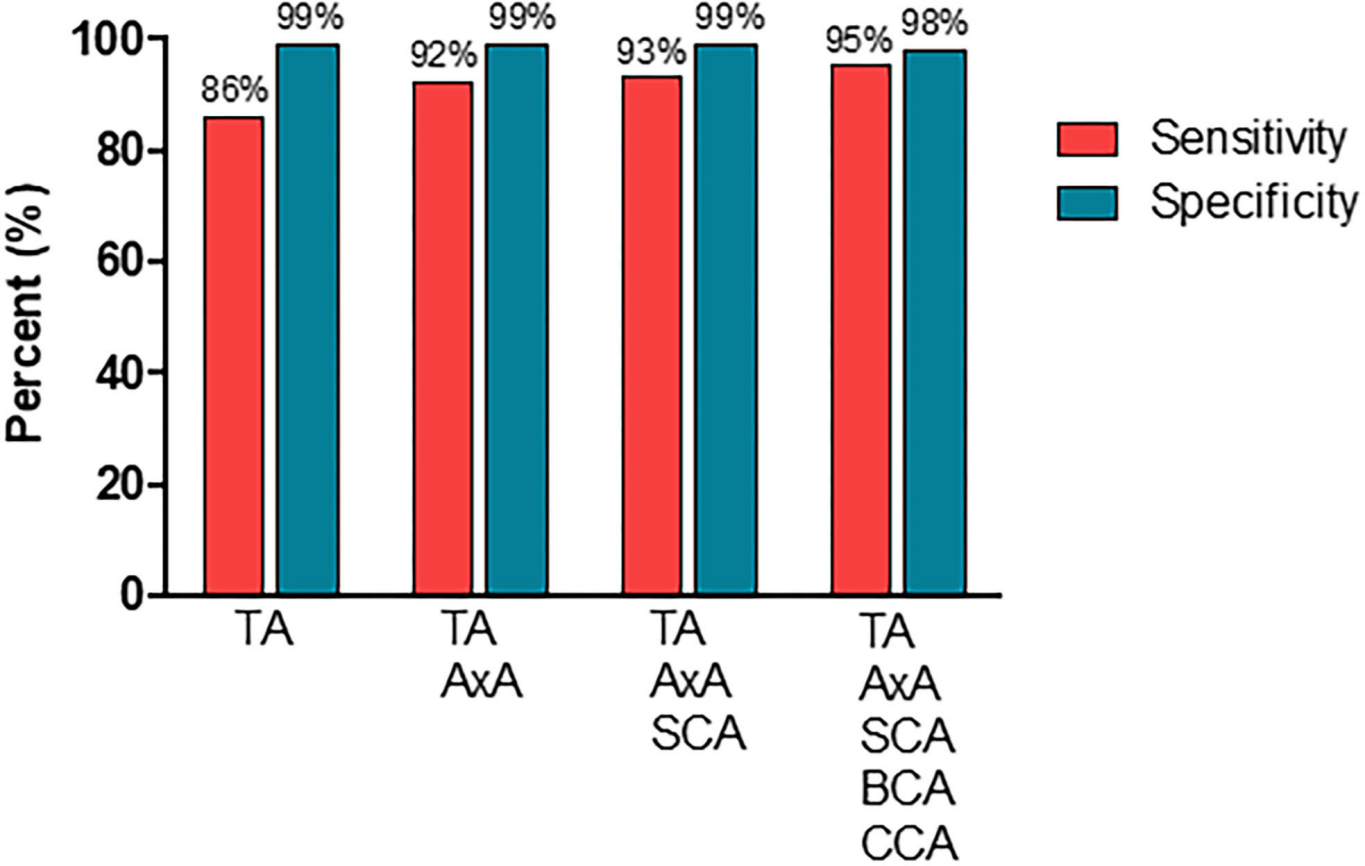
The diagnostic performance of CDU examinations in different vascular segments. TA, temporal artery; AxA, axillary artery; SCA, subclavian artery; BCA, brachiocephalic artery; CCA, common carotid artery.

Figure 4 display the diagnostic accuracy of the extended ultrasound protocol. With the clinical GCA diagnosis at ≥6 months as reference, CDU evaluation of temporal arteries yielded a diagnostic sensitivity and specificity [95% confidence intervals (CI)] of 86% (76–92%) and 99% (95–99%), respectively. By adding CDU examinations of the axillary artery, the sensitivity increased to 92% (83–97%) while the specificity remained at 99% (95–99%). Further incorporation of the subclavian artery in the diagnostic evaluation yielded a sensitivity and specificity of 93% (84–97%) and 99% (95–99%). Finally, by also including the brachiocephalic and common carotid artery in the CDU examination resulted in a sensitivity of 95% (88–99%) and a specificity of 98% (94–99%). Based on a prevalence of 41% in the present study population, our extended CDU protocol demonstrated a positive and negative prediction value of 97% (90–99%) and 97% (92–99%) respectively, and an accuracy of 97% (94–99%).

## Discussion

The present study confirmed our hypothesis that a CDU examination including temporal, axillary, subclavian, brachiocephalic, and common carotid arteries increased the diagnostic sensitivity for GCA without substantially affecting the specificity.

EULAR recommends ultrasound of temporal arteries, applied by trained ultrasonographers, as the first imaging modality in patients with suspected cranial GCA (6), and if the examination is negative or indecisive, a further evaluation of axillary arteries is suggested (6). The reason why the examination needs to be expanded in some cases is that some patients do not show any changes in the temporal arteries. However, which vessels to include, and the effects of the additional CDU scanning on the sensitivity and specificity of the examination remains unclear. The present study included 201 patients of which 83 had a confirmed clinical diagnosis of GCA 6 months after the ultrasound examination. Of these 83 patients, 48 (58%) displayed abnormalities solely in temporal arteries, whereas 23 (28%) showed signs of inflammation in both the temporal and extra cranial arteries, and 8 (10%) demonstrated abnormalities restricted to extra cranial vessels. Inflammatory changes were most frequently seen in temporal (86%), axillary (27%) and common carotid arteries (25%). Previous studied have shown somewhat conflicting results regarding the extra diagnostic value of incorporating other vascular territories than temporal arteries in the ultrasound examination. Schmidt et al. found that 20 of 53 patients with extra cranial GCA had no temporal artery abnormalities on ultrasound (20). No sensitivity or specificity calculation was presented since only GCA patients were included. Subsequent reports by Hop et al. demonstrated an increased sensitivity of 19% when adding the axillary to the investigation (21). However, other reports suggest that the increase in sensitivity may be only 2% (8), and the TABUL study found that only 2.4% of suspected GCA cases displays ultrasound abnormalities in axillary arteries (22). The reason for these discrepancies is not obvious, but differences in inclusion criteria between patients with and without cranial symptoms may have contributed. In the present study, all patients with suspected GCA were examined with an extended ultrasound protocol. We found that, by adding the axillary artery, five additional patients (6%) with GCA were identified. This increased the sensitivity with 6%, i.e., from 86% (95% CI, 76–92%) for ultrasound of only temporal arteries to 92% (95% CI, 83–97%) for temporal and axillary arteries. The specificity of CDU examination of only temporal arteries was 99% (95% CI, 95–99%), which is similar to recent a meta-analysis (10). The specificity remained essentially unchanged when adding the axillary artery to the examination.

Recently, a Halo Score have been developed to quantify the extent of inflammation detected with ultrasound (14). This score includes temporal as well as axillary arteries, and a higher score support GCA diagnosis (14). A modified Halo Score, also including subclavian arteries, has been suggested to better cover large vessel GCA, which is otherwise at risk of being missed (9).

We only identified one individual with detectable subclavian inflammation in which no vasculitic changes were observed in temporal or axillary arteries. Thus, by adding subclavian arteries to the ultrasound examination, the sensitivity increased by only one percent and the diagnostic accuracy was not obviously improved by including the subclavian artery if the axillary artery is incorporated in the examination protocol. It should also be noted that the above-mentioned patient with inflammatory signs in subclavian arteries also demonstrated changes in the brachiocephalic artery and common carotid arteries. These vessels are included in our extended ultrasound protocol (11, 16). Apart from the above-mentioned patient, two additional patients with confirmed GCA 6 month after the CDU examination were found by examining brachiocephalic artery and common carotid arteries. Thus, the sensitivity of diagnosing GCA with our extended protocol was 95% (95% CI, 88–99%).

It is important to note that other pathological conditions, e.g., malignancies, infections, and other rheumatologic diseases, may manifest with signs of inflammation in the vessel wall and thus mimic GCA (11, 23). In the present study, CDU showed an atypical inflammatory appearance in the brachiocephalic artery and common carotid artery in one patient. This patient was later diagnosed with chronic myelomonocytic leukemia. Thus, it must be emphasized that although ultrasound is an important tool in diagnosing GCA, it ought to be used as a complement to clinical history, laboratory results, and physical examination. It should also be underlined that although atherosclerosis can be distinguished from vasculitis it is far less common in temporal and axillary arteries compared to carotid arteries. Thus, atherosclerosis can disturb the sonographic evaluation of arteritis especially in other arteries than temporal and axillary arteries (18, 24). We found that the majority of patients demonstrated plaque in the carotid bifurcation or in three or more vessels. Although patients with GCA displayed higher IMT in large vessels, this was not the case with the brachiocephalic artery. This reinforce the notion that IMT should be interpreted with caution when differentiating between arteritis and atherosclerosis. Intimal medial thickness can be strongly affected by atherosclerosis and it is possible that an IMT above the cut-off is not related to GCA. Likewise, an IMT below the cut-off may be related to GCA (24). It is thus important that IMT and vessel wall appearance are interpreted in relation to each other. Nevertheless, the rather high frequency of atherosclerosis did not seem to affect the specificity for our extended protocol which was 98% (95% CI 94–99%), compared to a specificity of 99% (95% CI 95–99%) for examinations including temporal and axillary arteries.

Immediate treatment is recommended in patients with suspected GCA due to potential irreversible ischemic complications (1, 4, 5). However, as many patients are older and have other co-morbidities it is important to restrict unnecessary use of steroids due to its side effects. The present study demonstrated a good diagnostic accuracy using ultrasound examination of temporal and axillary arteries as recommended by the EULAR guidelines (6). However, if the examination is negative or indecisive, our results suggest that a further evaluation with our extended ultrasound protocol is of value.

A major strength of the study is the Swedish healthcare system, which is public, tax funded, and offers universal access. This significantly reduces the risk of selection bias and ensures a high coverage of cases. Potential drawbacks with this study were the retrospective design and that the examinations were performed as routine health care studies by more than one ultrasonographer. Interobserver variability was not measured. However, the strength of the extended CDU protocol is that it is strictly standardized, and thus, the results mirror real life in the routine health care system. Positron emission tomography/computed tomography (PET/CT) was conducted only in cases where it was clinically indicated. As such, no standardized comparison could be performed between PET/CT and CDU in patients with extra-cranial GCA.

## Conclusions

Ultrasound examination as the initial diagnostic test in patients with suspected GCA demonstrated a high sensitivity and specificity for the final diagnosis of GCA, both for cranial and extra-cranial GCA. The present study suggests that if no decisive abnormalities are detected in temporal or axillary arteries, an extended examination of neck arteries is indicated in order not to miss GCA involving other parts of the vascular tree. Finally, as the extended CDU protocol provides information about the general burden of inflammation it may also have a role in future monitoring of disease activity.

## Data Availability Statement

The raw data supporting the conclusions of this article will be made available by the authors, without undue reservation.

## Ethics Statement

The studies involving human participants were reviewed and approved by the Regional Ethical Board in Linköping (ref. 2013/33-31). Written informed consent for participation was not required for this study in accordance with the national legislation and the institutional requirements.

## Author Contributions

PE and HZ contributed to conception and design of the study. JS, PE, CSj, and HZ organized the database. JS performed the statistical analysis and wrote the first draft of the manuscript. CSv, PE, CSj, and HZ wrote sections of the manuscript. All authors contributed to manuscript revision, read, and approved the submitted version.

## Funding

This work was supported by grants from the Swedish Rheumatism Association (CSj), the Region Östergötland (ALF Grants; HZ and CSj), and the Gustafsson Foundation (CSj).

## Conflict of Interest

The authors declare that the research was conducted in the absence of any commercial or financial relationships that could be construed as a potential conflict of interest.

## Publisher's Note

All claims expressed in this article are solely those of the authors and do not necessarily represent those of their affiliated organizations, or those of the publisher, the editors and the reviewers. Any product that may be evaluated in this article, or claim that may be made by its manufacturer, is not guaranteed or endorsed by the publisher.
