# Supplementary material for: The Diagnostic Performance of an Extended Ultrasound Protocol in Patients With Clinically Suspected Giant Cell Arteritis
Source: Front Med (Lausanne). 2022 Jan 18;8:807996. doi: 10.3389/fmed.2021.807996 (PMC8804250; doi:10.3389/fmed.2021.807996)
Supplement: Supplementary file 1 [file Table_1.DOCX]

**Supplementary Tables**

**Supplementary Table 1.** Final diagnoses in patients without giant cell arteritis (GCA).

| **Final diagnosis** | **No. of patients (n=118)** |
| --- | --- |
| Polymyalgia rheumatica | 32 (27.1%) |
| Non-specific headache | 11 (9.3%) |
| Infection | 11 (9.3%) |
| Rheumatoid arthritis | 8 (6.8%) |
| Systemic inflammatory response | 6 (5.1%) |
| No clear diagnosis | 4 (3.4%) |
| Tension headache | 3 (2.5%) |
| Temporomandibular disorder | 2 (1.7%) |
| Spondyloarthritis | 2 (1.7%) |
| Oligoarthritis | 2 (1.7%) |
| Migraine | 2 (1.7%) |
| Idiopathic pericarditis | 2 (1.7%) |
| Fever | 2 (1.7%) |
| Arthritis | 2 (1.7%) |
| Uveitis | 1 (0.8%) |
| Stroke | 1 (0.8%) |
| Q-fever | 1 (0.8%) |
| Pyometra | 1 (0.8%) |
| Pyelonephritis | 1 (0.8%) |
| Psoriatic arthritis | 1 (0.8%) |
| Primary Sjögren’s syndrome | 1 (0.8%) |
| Polyarthritis | 1 (0.8%) |
| Myelodysplastic syndrome | 1 (0.8%) |
| Myalgia with positive anti-CCP | 1 (0.8%) |
| Musculoskeletal disorder | 1 (0.8%) |
| Multiple myeloma | 1 (0.8%) |
| Lung cancer | 1 (0.8%) |
| Kidney failure | 1 (0.8%) |
| Hip arthritis | 1 (0.8%) |
| Glomerulonephritis | 1 (0.8%) |
| Gingivitis | 1 (0.8%) |
| Fibromyalgia syndrome | 1 (0.8%) |
| Cryptogenic organizing pneumonia | 1 (0.8%) |
| Cluster headache | 1 (0.8%) |
| Chronic myelomonocytic leukemia | 1 (0.8%) |
| Chronic mastoiditis | 1 (0.8%) |
| Chronic headache | 1 (0.8%) |
| Central retinal vein occlusion | 1 (0.8%) |
| Mammary cancer | 1 (0.8%) |
| Aortitis | 1 (0.8%) |
| Amaurosis fugax | 1 (0.8%) |
| Adrenal insufficiency | 1 (0.8%) |

GCA, giant cell arteritis; ESR, erythrocyte sedimentation rate; CCP, cyclic citrullinated peptide.
